# Modeling impacts of climate change on the potential habitat of an endangered Brazilian endemic coral: Discussion about deep sea refugia

**DOI:** 10.1371/journal.pone.0211171

**Published:** 2019-05-21

**Authors:** Umberto Diego Rodrigues de Oliveira, Paula Braga Gomes, Ralf Tarciso Silva Cordeiro, Gislaine Vanessa de Lima, Carlos Daniel Pérez

**Affiliations:** 1 Programa de Pós-Graduação em Ecologia, Universidade Federal Rural de Pernambuco, Recife, PE, Brazil; 2 Programa de Pós-Graduação em Biologia Animal, Universidade Federal de Pernambuco, Recife, PE, Brazil; 3 Departamento de Biologia, Universidade Federal Rural de Pernambuco, Recife, PE, Brazil; 4 Centro Acadêmico de Vitória, Universidade Federal de Pernambuco, Vitória de Santo Antão, PE, Brazil; Harran University, Sanliurfa, Turkey, TURKEY

## Abstract

Climate and environmental conditions are determinant for coral distribution and their very existence. When changes in such conditions occur, their effects on distribution can be predicted through species distribution models, anticipating suitable habitats for the subsistence of species. *Mussismilia harttii* is one of the most endangered Brazilian endemic reef-building corals, and in increasing risk of extinction. Herein, species distribution models were used to determine the present and future potential habitats for *M*. *harttii*. Estimations were made through the maximum entropy approach, predicting suitable habitat losses and gains by the end of the 21st century. For this purpose, species records published in the last 20 years and current and future environmental variables were correlated. The best models were chosen according to the Akaike information criterion (AIC) and evaluated through the partial ROC (AUCratio), a new approach which uses independent occurrence data. Both approaches showed that the models performed satisfactorily in predicting potential habitat areas for the species. Future projections were made using the International Panel on Climate Change (IPCC) scenarios for 2100, with different levels of greenhouse gas emission. Representative Concentration Pathways (RCPs) were used to model the Future Potential Habitat (FPH) of *M*. *harttii* in two different scenarios: stabilization of emissions (RCP 4.5) and increase of emissions (RCP 8.5). According to the results, shallow waters to the south of the study area concentrate most of the current potential habitats for the species. However, in future scenarios, there was a loss of suitable areas in relation to the Current Potential Habitat (RCP 4.5 46% and RCP 8.5 59%), whereas there is a southward shift of the suitable areas. In all scenarios of FPH, the temperature was the variable with the greatest contribution to the models (> 35%), followed by the current velocity (> 33%) and bathymetry (>29%). In contrast, there is an increase of deep (50–75 m) suitable areas FPH scenarios, mainly in the southern portion of its distribution, at Abrolhos Bank (off Espirito Santo State). These deeper sites might serve as refugia for the species in global warming scenarios. Coral communities at such depths would be less susceptible to impacts of climate change on temperature and salinity. However, the deep sea is not free from human impacts and measures to protect deeper ecosystems should be prioritized in environmental policies for Brazilian marine conservation, especially the Abrolhos Bank, due to its importance for *M*. *harttii*.

## Introduction

Coral reefs are one of the most ecologically valuable ecosystems on earth [1] providing a number of ecosystem services [2], such as shelter for associated fishes [3] and crustaceans [4, 5, 6, 7], also serving as substrate for coralline algae [8, 9]. Stable water conditions are determinant for the maintenance of living corals on reefs [10]. However, effects of climate changes put at least 50% of shallow-water species in critical risk of extinction in the next 20 years [11, 12].

In the Southwestern Atlantic, coastal reef communities occur along of 3000 km of the Brazilian coastline [13], showing high endemism of reef-building species [14]. Four of those endemic species belong to the genus *Mussismilia*, commonly known as brain-corals [15, 16, 17]. Although molecular assessments on *Mussismilia* are still rare [18], the distinctiveness among species is well established, allowing rapid identification in the field [15]. The genus has at least two species in risk of extinction: *M*. *braziliensis* and *M*. *harttii* [19]. The first is restricted to shallow reefs of Bahia State and Abrolhos reefs, whereas the latter is found from the coast of Ceará to Espírito Santo States (from -3.822 to -18.0480 latitude).

*Mussismilia harttii* is the main reef-building coral in northeastern Brazil [14], usually found at depths of 2–6 m, with isolated records up to 80 m [20]. It is a hermaphrodite spawner species, with an annual reproductive cycle, releasing its gametes between September and November new moons [21]. Currently, *M*. *harttii* shows the lowest percentages of coverage among its congeners [22] and populations in severe decline [19]. Although its conservation status at the IUCN (International Union for Conservation of Nature) database is still regarded as “Data Deficient” (DD). however the “Red Book of the Brazilian Endangered Fauna” (2014, 2018), already classifies the species as EN (Endangered) [19].

The distribution of marine organisms, including corals, is determined by interactions of physical, chemical and biological factors [23]. Based on that, Species Distribution Models (SDMs) approaches can provide information on the potential distribution of species within specific study areas [24]. SDMs associate environmental or spatial data to a set of distributional information’s, such as distribution records [25], adopting the general thesis that the best indicator of a species climatic requirements is its current distribution [26]. Based on that, models indicate the environmental conditions in which a given species may occur [27], also indicating the most suitable areas for its occurrence [28, 29]. Modern SDMs studies began with BIOCLIM (the first SDM package), which became available in January 1984 [30] and are broadly applied to: prevent marine bioinvasions [31], conservation management planning [32], and especially to studies on climate changes [33, 34], predicting possible shifts on geographical distributions of key species [35].

The SDMs also can be used to calculate the relative adequacy of a given habitat occupied by a species and to estimate changes in such suitability over time [36]. In the present study, we applied SDMs to generate maps of Current Potential Habitat (CPH) and Future Potential Habitat (FPH) for *M*. *harttii* by the end of the 21st century. These maps indicate potentially suitable areas and estimate habitat gains and losses in the different climatic scenarios projected. The projections will serve as tools for management plans and reef conservation in the southwestern Atlantic reefs.

## Materials and methods

### Study area

The choice of the study area was based on a heuristic structure called **BAM** [37]. **B** represents the population dynamics (competition, predation, dispute over food or area) in the area where biotic parameters are suitable for the species. Estimation of these parameters would require a dense set of observations over large spatial extensions [38]. So we decided to neglect the effects of these biotic interactions on the modeling process. **A** represents mainly the abiotic conditions that do not depend on the presence or abundance of the species [39]. This geographic region is the fundamental niche of the species, which can potentially be invaded when both conditions are adequate (**A ∩ B**) but the species has not yet been able to reach is represented by **G’** [38]. It defines the dispersal potential of a species if the barriers are removed [40]. The parts of the world that have been accessible to the species via dispersal over relevant periods are symbolized by **M** [40]. The subset of the fundamental niche that is actually represented on relevant landscapes (**A** ∩ **M**) is the occupied area and can be defined as the existing fundamental niche [41]. Finally, this heuristic scheme then states that stable populations of a species will be found only in the region of intersection of **B**, **A** and **M**, (**B** ∩ **A** ∩ **M**) [37].

Barve et al. [40] emphasized the importance of **M** as the appropriate region through which models should be calibrated. If the fundamental niche extends beyond the environment boundaries represented in **M,** there may be truncation, which sub-characterizes niches and lead to different sets of problems for model transfer [42]. Increasing the extent also often includes absences that are more distant environmentally from the presences, but due to the limitations of **M**, makes the model look better than it actually is [40]. Using a larger study region (**G**') would prone the model to overfit environmental conditions present in the region where the species is known to occur, in situations referred as non-equilibrium distributions [43].

According to Owens et al.[42], models applied to centrally occurring species within **M** environments should not present problems with extrapolation, even in the presence of new conditions within a transfer region (**G’**). This may produce more realistic predictions of the potential distribution of a species [42].

**Fig 1:**
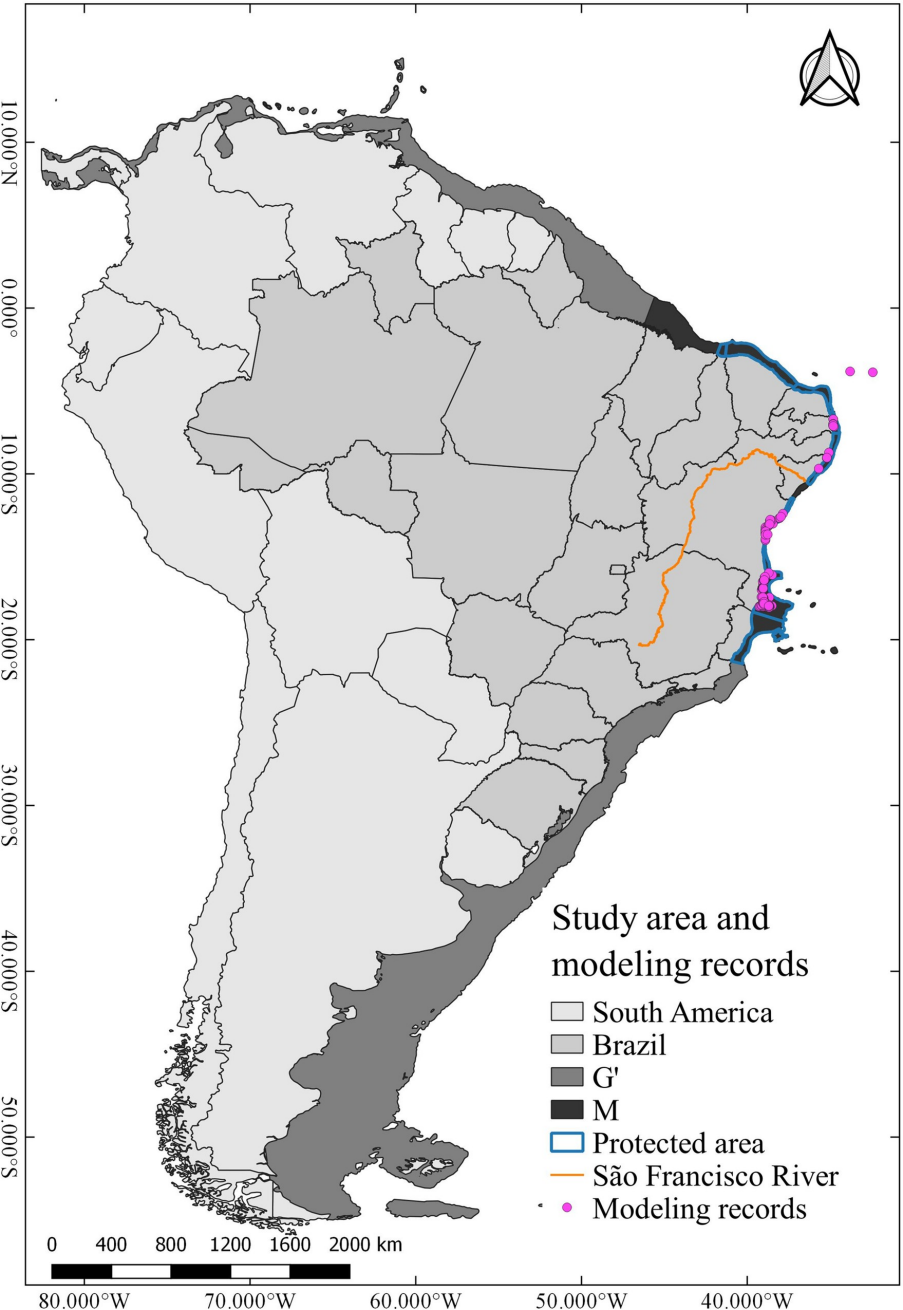
Map of the study area and occurrence records. Study area, including **G’** region (Southeast South American coast up 100 m), **M** region (calibration area), priority conservation area for *Mussismilia harttii* and modeling records (one point in each pixel of 1 km^2^).

We divided the study area into two regions: The first one (**M**) is the area containing all occurrence records of the species and also includes the priority areas for its conservation, according to the Brazilian Ministry of the Environment (Portaria N° 19, of March 9, 2016—ICMBio). **M** extends between the states of Ceará and Espírito Santo in Brazil [37, 40]. The second region (**G’**) comprises the entire coastal zone of the Southwestern Atlantic Ocean, from the intertidal zone down to 100 m deep [44]. The model was calibrated in **M** and the potential habitat for the late 21st century was projected in **G'** (Fig 1).

### Occurrence records

An extensive search was made in specialized literature (using the terms "Mussismilia", "harttii", "Brazil", and "Brasil"), with publications containing precise geographic information (latitude, longitude and/or georeferenced maps). Occurrence records with errors or lacking georeferenced were not used in the analyzes, resulting in a set of 147 records which were used to discuss the suitable areas provided by the models. The search focused on records of *M*. *harttii* from the last two decades, to reduce the loss of quality of data due to the action of time and dynamism in natural systems [45]. Sometimes, these data may be influenced by drastic phenomena, such as the local extinction of species [46] or changes in its distribution and abundance patterns [47]. Sampling bias on occurrence data is also common in more accessible areas (more studied) because of regional interests [48]. This may reduce the model's ability to predict independence of the spatial data [49]. We used SDMtoolbox [50] to filter occurrence data by environmental heterogeneity. This gradual filtering method is useful in studies with reduced numbers of occurrences and also avoids data with habitats with habitats other than the known occurrence of a species. [50]. We calibrate the filter with radius of 5 km^2^ for the raster of environmental heterogeneity, three numbers of heterogeneity of classes, maximum distance 25 km and minimum 5 km. At the end of the process, we obtained 102 (S1 Appendix) occurrence records to calibration model (CPH) and projection (FPH). These filtered occurrence records were called modeling records (Fig 1).

Species data collected *in situ*, from reefs located in the States of Paraíba, Pernambuco, Alagoas, Bahia, as well as independent species presence data, were not used during the modeling process calibration model (CPH), but *a posteriori* to evaluate the model. These occurrence records were filtered using the same procedure of the previous step. Finally, we obtained 33 filtered and independent records of the modeling records (S1 Appendix). Validation with data independent from the model ensures greater robustness in its evaluation [51]. These occurrence records are called evaluation records.

### Selection of environmental layers

The environmental characterization variables provided by Bio-oracle (available at http://www.bio-oracle.org) were used. This global database provides *in situ* current and satellite-based oceanic information of the surface and seabed in a 30 arc seconds resolution (~ 1 km^2^) [23]. Bio-oracle also provides future variables based on the projections made by the International Panel on Climate Change (IPCC) for 2100 [52], in scenarios with different concentration levels of greenhouse gases [53]. In this study, we used the benthic Bio-oracle variables that were produced with an interpolation process, considering the geographic position and the depth of the cells, as inferred from a bathymetric layer.

Projections of the IPCC for 2100, developed by different research groups [54, 55], provide likely ranges of global temperatures in future scenarios according to population, economic growth and carbon use. These projections, called Representative Concentration Pathways (RCPs) [44], were used to model the FPH for *M*. *harttii* in two different scenarios: stabilization of emissions (RCP 4.5) and increase in emissions (RCP 8.5) [56, 57].

To ensure model transfers for the future, we focused on the calibration and extrapolation of models in climate variables [58]. These climate models are defined as part of the fundamental ecological niche or "climatic niche", predicting the potential extent of organisms in altered climate, but do not considering the dispersion of species [59]. It is possible to couple bioclimatic envelope models to dispersion simulations [60]. In this study, we incorporated the current velocity variable provided by Bio-oracle for 2100 and the oceanic bathymetry provided by Natural Earth (available at http://www.naturalearthdata.com), which is a limiting factor for the dispersion and resilience of the species [20].

The number of variables used may depend on the number of occurrence records [61], and when there are few records, such as endemic or threatened species, a small number of variables may be sufficient [62].

We submit these variables to two PCAs (supplementary material) to identify: (1) which variables have greater importance to the model (r > 6), excluding those with little relevance (r < 6), and leaving a set of seven climatic variables (S2 Appendix); (2) how many variables, from the seven remaining, explain 100% of the environmental space of **M**. The second PCA showed that it is possible to explain more than 99% of the environmental space of **M** using only three variables (S2 Appendix).

We used Mobility-Oriented Parity (MOP), available at the “ntbox” package [63], to identify areas of strict extrapolation and also to calculate the environmental similarity between the calibration and projection regions (**M** and **G’**) [42]. Only five of the seven climatic variables available at Bio-oracle had environmental similarities between **M** and the same region of **M** in **G’**.

Finally, we add the bathymetry to the five remaining environmental variables and grouped these six variables into 14 sets, each with three variables (Table 1) that had no correlation [64] greater than 7.9 with each other (S2 Appendix), all ecologically or physiologically relevant [48].

**Table 1:** Details about the five variables used in the modeling process.

Set	Variables			Set	Variables		
1	temp_max	veloc_lt_min	salin_range	8	temp_range	temp_max	bathymetry
2	temp_range	veloc_lt_min	salin_range	9	temp_range	temp_max	veloc_lt_min
3	temp_range	veloc_mean	salin_range	10	temp_range	temp_max	veloc_mean
4	temp_max	veloc_mean	salin_range	11	temp_max	veloc_mean	bathymetry
5	temp_max	bathymetry	salin_range	12	temp_max	veloc_lt_min	bathymetry
6	temp_range	bathymetry	salin_range	13	temp_range	veloc_lt_min	bathymetry
7	temp_range	temp_max	salin_range	14	temp_range	veloc_mean	bathymetry

Table 1. Environmental variables grouped 14 sets used to construct the models for Current Potential Habitat of *Mussismilia harttii*. Abbreviations: temp_max (maximum temperature), temp_range (temperature range), veloc_lt_min (long-term minimum mean current velocity), veloc_mean (mean current velocity) and salin_range (salinity range).

### Modeling process approach

The maximum entropy approach MaxEnt v. 3.3.3 [65, 66, 67] was used to model the potential distribution of *M*. *harttii*. MaxEnt is one of the most widely used algorithms for SDMs [68], because it presents consistent predictive performance compared to other algorithms [69], especially when the number of occurrence points is low [51, 70]. Maxent also resolves truncation issues via a more conservative assumption that is termed ‘clamping’. When a pixel has a value for a given variable outside the range covered by the model (calibration model), is given to that pixel the closest value of the pixel present for that variable in the model [43].

Traditionally, the task of choosing the best parameter values has been considered a challenge for these models [71]. We used the "ENMeval" package [72] to choose the best parameter values. For each set of variables, 48 models were constructed [73] through the dismo package [74], using the three variables of each set, modeling records (using checkerboard2 for partitioning of occurrence data), maximum background number (10000) and using the following parameter settings: multiplayer regularization values (0.5–4.0 with 0.5 intervals), six resource class configurations: L, LQ, H, LQH, LQHP and LQHPT (where L = linear, Q = quadratic, H = hinge, P = product and T = threshold). The Jackknife function of MaxEnt [75] was used to identify the percentage of contribution for each variable. The best model for each set of variables (Table 1) was chosen based on the lowest Akaike Information Criterion (AICc) values [76]. The Akaike information criterion is an important metric in ecological niche modeling [77], but it is necessary to use other statistical criteria to evaluate the performance of the model through independent data [78].

### Evaluation of the models

The Area Under the Receiver Operating Curve (AUC-ROC) is the most common metric to evaluate the accuracy of models [79]. AUC values ≤ 0.5 indicate that the model failed to perform better than random expectations, whereas values close to 1 indicate a good performance of the model [80]. In practice, the AUC-ROC is calculated based on a series of trapezoids [81], with the curve essentially "connecting the points" representing the different thresholds of the prediction [82]. This approach is used when input data is partitioned, in this case into training and test data [83]. When biotic data are divided into presence and absence (background), the AUC measures the discriminatory ability of the model to correctly predict the origin of these data if randomly selected [51].

Although the use of AUC-ROC for model evaluation is not questioned herein [84], we additionally used the partial ROC (AUCratio) to choose the best model. AUCratio is an independent cutoff threshold metric where significant values are above 1 [85]. The AUCratio is a ratio between the predicted model AUC and null expectation [82] that a model generated with random data does not have a better prediction than the models generated with the input data [86]. We calculated the ratio of AUCrandon (at level of 0.5) and the AUCatual (calibrating 5% of omission and 1000 bootstrap interactions) using the predicted distribution model [68] and evaluation records, through the package "ntbox" v.0.2.5.3 [63] for Rstudio [87], to ensure greater robustness in model analysis [88].

The best model was designed for the two future scenarios (RCP 4.5 and RCP 8.5), within the **G’** region, through the "predict" function available in the dismo package [74].

### Suitability area

Based on threshold values (S3 Appendix), the continuous maps of CPH and FPH were transformed into binary maps of suitability or probability [89], in which pixels are classified as "adaptive / presence" and "non-adaptive / absence" [51]. Through the maps with the presence pixels, we calculated the total area, the lost, gained and maintained areas. Additionally, we also calculated those areas in the depths of 0 to -20, -20 to -50, -50 to -75, and—75 to -100 meters. All area analyzes (CPH and FPH) were developed in the **M** region.

## Results

The variables used to model CPH (set 12) were,: in decreasing order of contribution: long-term minimum mean current velocity (42.7%); bathymetry (31.9%) and maximum temperature (25.4%) (S3 Appendix).

The maximum training sensitivity plus specificity cloglog threshold used to generate the binary maps maximized the sensitivity and specificity of the model [90]. This threshold is best suited for studies on rare or endangered species [86], as it reduces the over-prediction rate and selects only areas with high environmental suitability [51]. The thresholds of CPH (0.314) and FPH (RCP 4.5–00.314 and RCP 8.5–0.241) show that a random prediction in a fraction of the same area does not have a better prediction than the points used in the test step [86].

**Fig 2:**
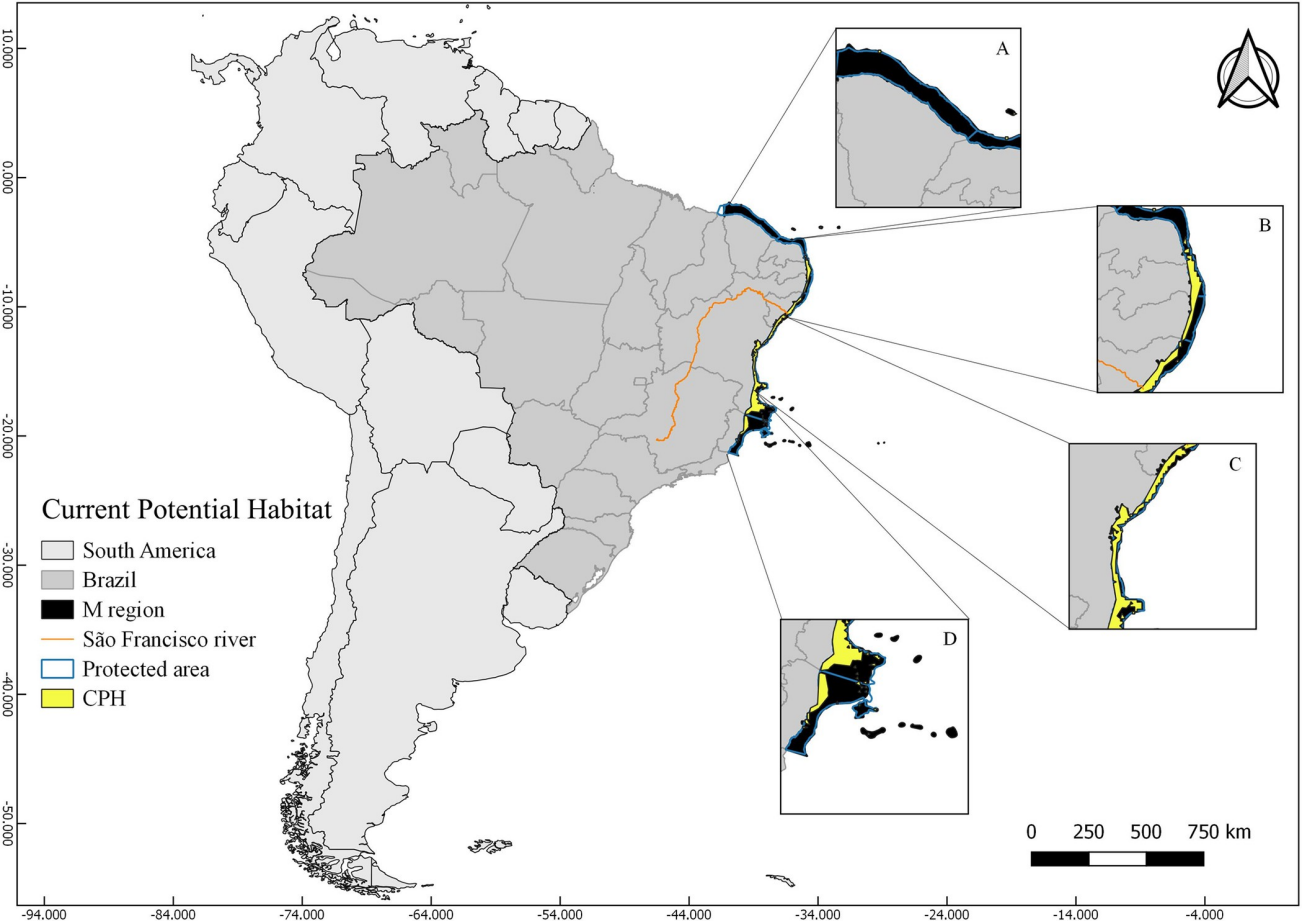
Map of current potential habitat (CPH) of *Mussismilia harttii*. Highlighted figures (A, B, C and D) show the different areas in the **M** region: A) Ceará and north of Rio Grande do Norte States; B) South of Rio Grande do Norte, Paraíba, Pernambuco and north of Alagoas States; C) South of Alagoas and north of Bahia States; D) south of Bahia and Espírito Santo States.

The CPH of *M*. *harttii* was constructed using the parameters LQHPT and 0.5 regularization multiplayer (S4 Appendix). It represents a suitable area corresponding to 0.276% of the **M** area (Fig 2; Table 2). The sites north of the São Francisco River show a smaller suitability (21.1%) (Fig 2A and 2B; Table 2), whereas the largest suitable areas are concentrated southwards the São Francisco River (77.9%) (Fig 2C and 2D; Table 2). The AICc (S4 Appendix) and AUCratio (S5 Appendix) of the model were 0 and 1.516141349, respectively.

**Table 2:** Areas of suitable habitats.

total area		north	south	New area		Kept area		Lost area	
CPH	52610	11431.2	41010.5	north	south	north	south	north	south
RCP 4.5	20712.3	222.8	20489.4	183.4	18323.2	39.4	2167.3	11391.7	38842.6
RCP 8.5	28378.2	807.8	27570.3	663.7	25664.6	144	1903.8	11287.1	39105.7

Table 2. Approximate values of current potential habitat areas (CPH) and future potential habitat areas (FPH) for *Mussismilia harttii* in two different scenarios of climatic projections for the year 2100 (RCP 4.5 and 8.5); including the new, lost and kept areas in each region: north and south of the São Francisco River.

**Fig 3:**
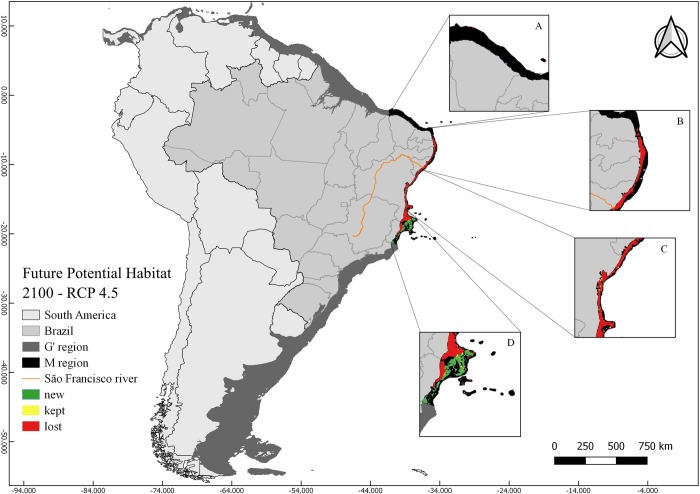
Map of future potential habitat (FPH) of *Mussismilia harttii* in a scenario of stabilization of greenhouse gas emissions (RCP 4.5) in the year 2100. FPH includes regions with kept, new, and lost suitability compared with the present (CPH). Highlighted figures (A, B, C, and D) show the different areas in the **M** region: A) Ceará and north of Rio Grande do Norte States; B) South of Rio Grande do Norte, Paraíba, Pernambuco and north of Alagoas States; C) South of Alagoas and north of Bahia States; D) south of Bahia and Espírito Santo States.

**Fig 4:**
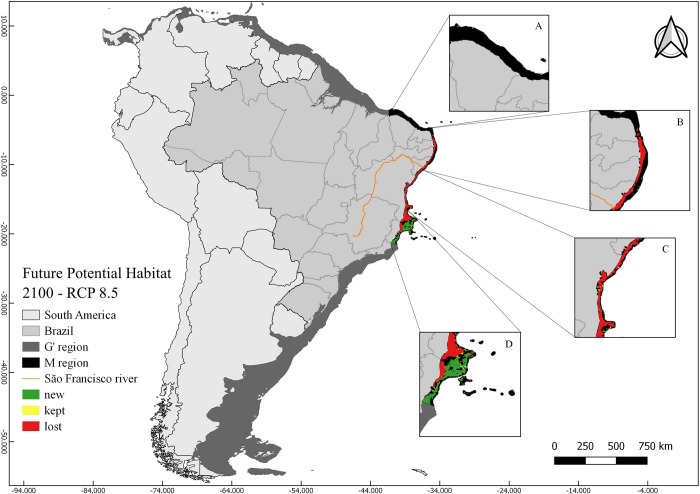
Map of future potential habitat (FPH) of *Mussismilia harttii* in a scenario of increase of greenhouse gas emissions (RCP 8.5) in the year 2100. FPH includes regions with kept, new, and lost suitability compared with the present (CPH). Highlighted figures (A, B, C, and D) show the different areas in the **M** region: A) Ceará and north of Rio Grande do Norte States; B) South of Rio Grande do Norte, Paraíba, Pernambuco and north of Alagoas States; C) South of Alagoas and north of Bahia States; D) south of Bahia and Espírito Santo States.

The two future distribution scenarios for *M*. *harttii* (RCP 4.5 and RCP 8.5) were characterized by the loss of suitable areas in relation to CPH in the **M** region (RCP 4.5 46% and RCP 8.5 59%) (Table 2). Although there were losses in the areas to the north and south of the Sao Francisco River, the reduction of the total suitable area was greater in the northern limit of the distribution, and the gain of new areas in the southern portion (Figs 3 and 4). In all FPH scenarios, the maximum temperature was the variable with the greatest contribution to the models (> 35%), followed by the current velocity (> 33%) and bathymetry (>29%).

In a scenario in which the emissions of greenhouse gases stabilize (RCP 4.5), the FPH of *M*. *harttii* represents a suitable area corresponding to 39.3% of the CPH (Fig 3; Table 2) (). The sites northwards of the São Francisco River have a smaller suitable area (1.1%) (Fig 3A and 3B; Table 2), while the largest areas of adequacy are concentrated southwards of the São Francisco River (98.9%) (Fig 3C and 3D; Table 2). The AICc (S4 Appendix) and AUCratio (S5 Appendix) of the model were 0 and 1.720858124, respectively.

In a scenario with increased greenhouse gas emissions (RCP 8.5), the FPH of *M*. *harttii* represents a suitable area corresponding to 53.9% of the CPH (Fig 4; Table 2) increasing 88% of the CPH). The sites north of the São Francisco River again shows a smaller area (2.9%) (Fig 4A and 4B; Table 2), whereas the largest suitable areas are concentrated southwards of the São Francisco River (97.1%) (Fig 4C and 4D; Table 2). The AICc (S5 Appendix) and AUCratio (S4 Appendix) of the model were 0 and 1.459161766, respectively.

Both current and future suitable areas for *M*. *harttii* are mostly within the Preservation Area for this species, with exception of Todos os Santos Bay, Bahia State (Figs 2, 3 and 4C). The two scenarios of future (year 2100) distribution of the species showed bathymetric expansion towards deeper areas, mainly in the southern portion of its distribution, with a latitudinal restriction by the loss of suitable areas in its northernmost limits (Figs 3 and 4; Table 2).

In the current scenario (CPH), ~ 54% of the suitable areas are shallower than 20 m deep, ~ 43% between 20–50 m, ~ 2% between 50–75 m and ~ 1% between 75–100 m (Table 3). In the two future scenarios (RCPs), > 40% of the new areas suitable for the species were concentrated between 20 m and 50 m and > 19% between 50 m and 75 m, mostly to the south of the São Francisco River (Table 3).

**Table 3:** Areas (km^2^) of suitable habitats by depth ranges.

	**Depth**	**0–20**	**20–50**	**50–75**	**75–100**								
**CPH**	**north (km**^**2**^**)**	**5827.6**	**3719.6**	**77.3**	**38.3**								
	**south (km**^**2**^**)**	**19653.1**	**16529.7**	**715.8**	**123.8**								
		**New areas (km**^**2**^**)**				**Kept areas (km**^**2**^**)**				**Lost areas (km**^**2**^**)**			
	**Depth (m)**	**0–20**	**20–50**	**50–75**	**75–100**	**0–20**	**20–50**	**50–75**	**75–100**	**0–20**	**20–50**	**50–75**	**75–100**
**RCP 4.5**	**North (km**^**2**^**)**	**72.3**	**47.1**	**18.8**	**5.4**	**0**	**0**	**1.1**	**1.1**	**5715.7**	**3648.4**	**73.7**	**36.9**
	**South (km**^**2**^**)**	**1218.3**	**12563.7**	**4071.6**	**142.6**	**462.7**	**1411.2**	**126.7**	**18.7**	**19212.8**	**15125.6**	**589.5**	**105**
**RCP 8.5**	**North (km**^**2**^**)**	**39.5**	**82.5**	**38.9**	**19.9**	**0**	**5.1**	**13.7**	**8.4**	**5715.7**	**3643.3**	**61.2**	**29.6**
	**South (km**^**2**^**)**	**2103.2**	**12346.6**	**9861.9**	**494.9**	**283.1**	**1310.2**	**158.6**	**16.8**	**19392.5**	**15226.4**	**557.5**	**106.9**

Table 3. Approximate values of current potential habitat areas (CPH) and future potential habitat areas (FPH) for *Mussismilia harttii* in two different projected climatic scenarios for the year 2100 (RCP 4.5 and RCP 8.5). North and south of the São Francisco River arranged in four depth ranges.

In summary, future scenarios show a loss of suitable areas for the persistence of the species in relation to CPH in the **M** region (RCP 4.5 46% and RCP 8.5 59%). There is also a prominent shift of suitable areas to the south of the **M** region (Figs 3 and 4). In contrast, there is a massive increment of suitable areas towards deeper waters (50–75 m) (1023% in RCP 4.5 and 1229% in RCP 8.5), mainly in the southern portion of its distribution, at the Abrolhos Bank (off Espirito Santo State) (Table 3).

**Fig 5:**
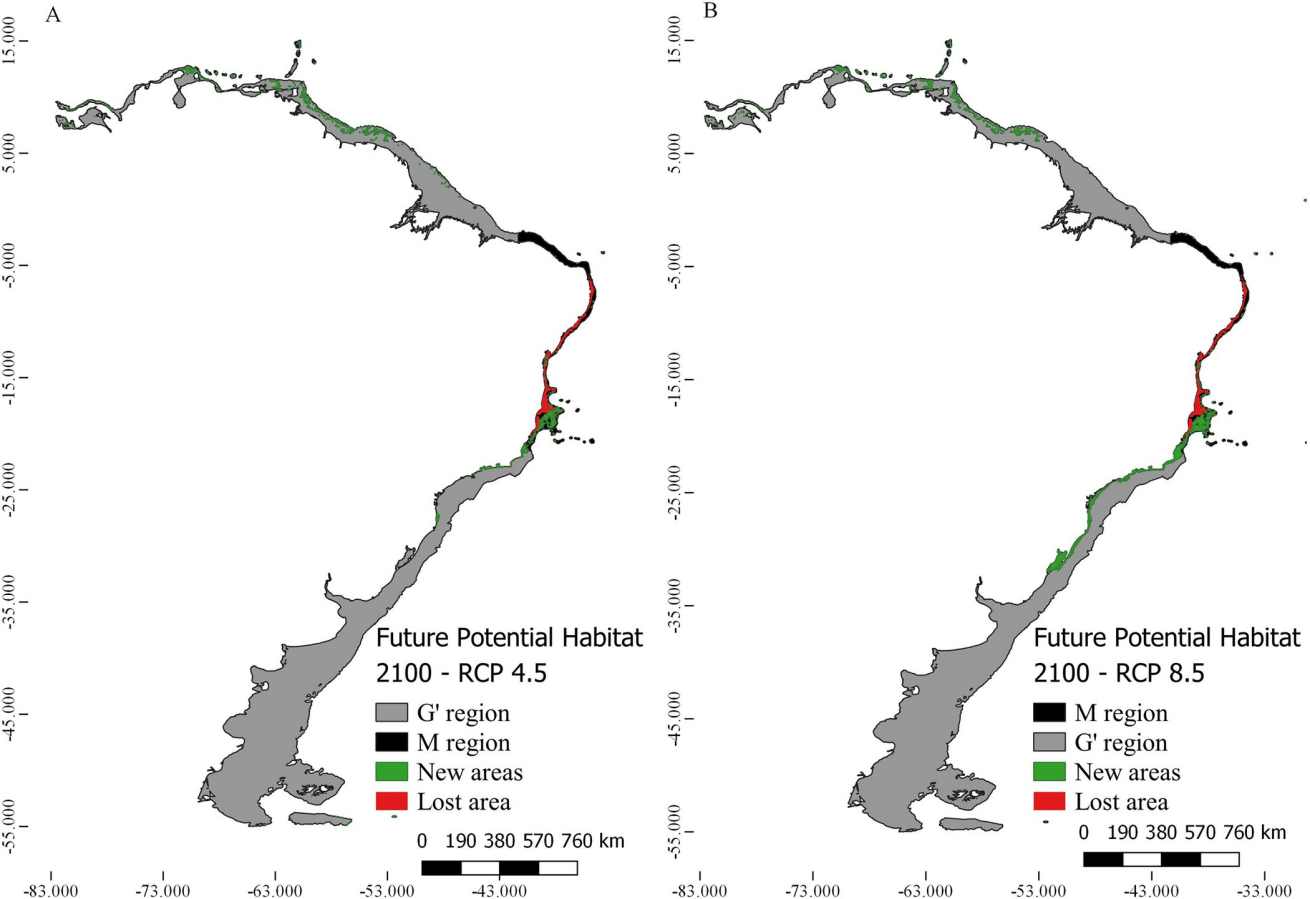
Map of future potential habitat (FPH) of *Mussismilia harttii* in two scenarios of greenhouse gas emissions (RCP 4.5 and RCP 8.5) in the year 2100. The map includes the area of study (**G'**), area (**M**), the FPH in the two future scenarios in the entire study area (**G'**) and the area lost in relation to CPH.

The two future scenarios showed areas with potential habitat outside of the **M** region. The FPH in the RCP 4.5 scenario has 44883.4 km^2^, whereas FPH in the temperature increase scenario (RCP 8.5) has 77694.3 km^2^ outside the area **M** (Fig 5). These potential areas outside the **M** region are located in the north of the Amazon River (North Brazil and Guianas) (up to14°N latitude) and in the southern Brazilian coast (up to 32°S latitude).

## Discussion

### Visualization and Interpretation of ecological niche models

Predicting and mapping potential suitable habitats for threatened and endangered species is critical for monitoring and restoring their natural populations [91]. In this sense, a modeling approach is an effective tool to predict the direction of contractions and expansions of species distribution [92], producing probability maps for the presence or relative suitability of a species [93].

Besides elevated CPH validation indexes, literature data (not geo-referenced and therefore not used in the model) also record *M*. *harttii* in areas indicated by the model as suitable for the species, such as the southern portion of the Abrolhos bank (Espírito Santo State) [94]. A model that fails to omit known points of presence is less accurate than those predicting unknown inhabited areas [95]. These unknown areas provide a precise representation of the spatial extent of habitable conditions for the species [82].

Although the FPH of the species (Fig 5) extends beyond the calibration area of the model (**M**), it was already expected an area of potential species habitat larger than the real distribution [65]. Consequently, projections beyond the time interval of a training dataset (distribution in future dates) require cautious interpretations to avoid possible misinterpretations [96]. Such caution is because AUC values tend to increase when the selected background area is larger than the observed current habitat of a species [97]. Although the AUC values (close to 1) showed that the models performed very well with the results [90, 91] (better than any model generated with a set of random predictors [83]), it was necessary to use a different approach to evaluate the models. In the AUC metric, the weight of commission errors is much lower than that of omission errors, which makes it an inappropriate performance measurement [98].

The AUCratio also showed a good performance of the model, with values above 1 [82] and close to 2. These results allowed us to evaluate the statistical significance of the AUC itself [98]. In this way, it is more appropriate to evaluate the model performances [84]. The thresholds used to generate the binary maps are best suited for applications in ecological niche templates [87] [39] by better predicting independent occurrence data [46] [99] [100].

### Environmental variables and *M*. *harttii’s* habitat

Even though the effects of each environmental variable on the population dynamics are unknown [101], the variables chosen to model the habitat suitability for *M*. *harttii* are in accordance with default conditions in previous studies on anthozoans [102].

Current Potential Habitats for *M*. *harttii* were mostly influenced by current velocity, bathymetry and temperature, respectively. Nutrient uptake in coral reefs is directly affected by current speeds [103], which shape their distribution in both shallow and deep waters [104]. The **M** area is under the influence of the Brazilian and the North Brazilian Current [105]. Studies on genetic connectivity with the genus *Mussismilia* (*M*. *hispida*) showed that these currents may consist in a barrier to coral dispersal [106]. In shallow reefs, currents cause constant sediment resuspension, which influences the coverage and composition of benthic organisms [107], changing the visibility of water [108], influencing the microbial community of corals [109] and the behavior of the coral larvae during the nesting phase [110]. Considering that most records of *M*. *harttii* result from shallow reefs, it is expected that the species is rather influenced by sediment resuspension. Despite Brazilian corals as a whole are resistant to the input of terrigenous sediments and to relatively turbid waters 101], *M*. *harttii* has preference for clear waters, in which it is more abundant [111].

Temperature, salinity and light have major effects on where reef-building corals grow [112]. Despite the temperature showed the lowest contribution for the CPH, it is undoubtedly determinant for the future persistence of coral species, as 50% of these corals are threatened by climate changes [11, 12]. Our results also show the importance of temperature in the FPH for *M*. *harttii*. This species suffers thermal stress in temperatures higher than 31.0°C, leading to long-term damage or death [113]. In fact, a recent study reported massive coral bleaching events in temperatures above 27° in Abrolhos reefs [114], which concentrate most records of *M*. *harttii* in the present study (Fig 1). Climate change-related increase of temperature will affect wind regimes, ocean circulation and, consequently, precipitation and continental runoff, directly affecting the salinity in coastal waters [115, 116]. That may explain the spatial reduction in shallow waters projected in the FPH, and the increase in deeper zones. However, such habitat shifting can be related to several factors.

Another important factor is the competition with algae (macroalgae and filamentous algae). These organisms dominate Brazilian benthic communities on reefs down to 15 m deep [117]. Algae are favored by anthropic impacts, such as reduction of herbivorous/grazer fishes by overfishing, and increase of land nutrient discharges [118, 119]. Thus, in future scenarios, algae will likely continue to be favored, and its competition with corals tends to reduce coverage of the later in shallow waters. In contrast, besides less light availability, deeper areas would be less susceptible to the influence of runoff, temperature and salinity changes. Despite the lack of earlier baselines for Brazilian benthic communities, it is possible to affirm that the current scenario is result of a sum of anthropic impacts, as studies back in the 1960’s describe distinctive zonation and coverage in these communities [120].

### Current distribution of *M*. *harttii*

Most of the current suitable distribution area for *M*. *harttii* (CPH) is south of the São Francisco river, where most published records are concentrated. Despite records in the coast of the Espírito Santo State (~ 19ºS) were not used in our analyses, that area is known as the southernmost distribution limit for the species [111], with the highest percentage of CPH. That region coincides with a center of diversity within the Brazilian Province (20°S to 23°S), as indicated for benthic organisms, such as algae, invertebrates and fishes [117, 121, 122, 123]. That center is favored by the confluence of currents in the Brazilian coast, creating a transition zone between tropical and subtropical diversity [117]. Despite a limited number of records of *M*. *harttii* and a smaller percentage of CPH to the north of the São Francisco River, the species is the main reef-builder northwards the São Francisco river [14].

Most records of *M*. *harttii* are from shallow reefs, between 2 and 6 meters [120] and consequently close to the coast. However, scattered records show this species occupying deeper reefs (up to 25 m) [94] and even at mesophotic depths [20]. Similarly, most of the CPH is concentrated in shallow waters (0–20 m), but with deeper suitable habitats commonly occurring, especially in the southern portion of species distribution. Most of current records of the species are within the Abrolhos Bank, in the East coast of Brazil (16°40′ – 19°40′S and 37°20′ – 39°10′ W), which harbour on of the most rich na extensive coral reef system in the South Atlantic [94, 124]. The area is composed by a mosaic of protected areas, as well as several distinct environments, such as biogenic reefs, euphotic and mesophotic reefs and rhodolith beds [94]. The records are mainly concentrated in the shallow areas of the bank, but some came from southern deeper reefs, between 12 and 25 m (Espírito Santo State), a less studied area [94].

### *Mussismilia harttii*’s response to climate change by the end of the 21st century

Future distribution models (RCP 4.5 and RCP 8.5) of *M*. *harttii* showed expansion of suitable areas, towards deeper sites where there are few records of this species. Concomitantly, there was a reduction of suitable shallow water areas, especially at the northeast distribution limit, which suffered the greatest losses (Figs 3 and 4). It is a more concerning fact if we consider that the species is the main reef-building coral in northeastern Brazilian reefs [14].

A recent study on *M*. *harttii* [125] estimates a decline of its populations in their current geographic range in shallow waters. Our results also indicate the same in a future scenario (RCP 8.5) with a loss of 98.5% of the current suitable area (~ 25480.7 km^2^ in shallow waters (0–20 m), concentrated mainly in the northeast distribution of the species (Table 3). Conversely, the results show a 1270% increase at deeper areas (50 m—75 m) in future scenario (RCP 4.5) (Table 3). Thus, in a future scenario, the species would lose suitable habitats in coastal shallow sectors, followed by a gain of deeper habitats, which could serve as refugia in face of climate changes, if other environmental conditions such as luminosity, salinity and others are favorable [112].

Areas with potential habitat for *M*. *harttii* outside the **M** region in the two future scenarios, such as the northern coast of South America and the southern coast of Brazil, may be inaccessible due to geographic barriers for coral dispersion. These include colder water masses in the region of Cabo Frio (Rio de Janeiro), to the south [16], and the plumes of São Francisco [126] and the Amazon rivers [127], to the north. In compensation, deeper potential habitats for *M*. *harttii* within the **M** region are mostly concentrated in the Abrolhos Bank, which might serve as refuge areas in future climate change scenarios.

### Deep sea refugia strategy

The “deep reef refugia hypothesis” (DRRH) considers that coastal anthropic impacts and thermal stress effects are progressively reduced with depth [128, 129]. Therefore, mesophotic coral ecosystems, between 30 and 150 m, have been treated as important refugia for shallow reefs diversity [130, 131], temporarily supporting coral populations from shallow-reefs under stress conditions [132]. Such areas would provide shelter in which these populations might persist in a long time [129], and from which would subsequently expand [133], recovering previously damaged areas [117, 134].

The reduction of shallow suitable areas and increase of deeper habitats suggest the potential of *M*. *harttii* for using mesophotic reefs as refugia, ensuring its subsistence. However, the DRRH is more adequate for species with wide depth distribution ranges [117] and presupposes larvae exchange between deep and shallow populations [135], which have been demonstrated to be local and species-specific [136]. Despite *M*. *harttii* is particularly representative in shallow waters (2–6 m), scattered records show this species occupying deeper reefs (up to 80 m) [20, 94, 137] (S6 Appendix), which reinforces the potential of the species to occupy deep mesophotic areas.

Even showing wide depth ranges, connectivity between coral populations is not always continuous along bathymetric gradients [134]. Consequently, it is still unknown if deeper populations of *M*. *harttii* would serve as genetic stocks for shallow waters, as most of its deep records are sparse and rare [135]. In any case, the expansion of deeper suitable areas may result in the expansion of deeper populations of *M*. *harttii*, regardless of the maintenance of coastal populations. In case of connectivity, such refugia would contribute for the recolonization of the coastal zone affected.

Studies using of global climate models mostly suggest that few shallow coral species will persist under a sea surface temperature increase of 2°C in the next one hundred years [138]. Nevertheless, given the current slowness in mitigation measures, it is expected an increase of 3.1°C in the same period (RCP 8.5) [139]. In such scenarios, identify and protect deep sea refugia must become priorities for species conservation [131], considering the various factors defining a potentially true refuge [140].

### Threats and perspectives for conservation

The main global threats to coral species are related with greenhouse gas emissions (RCP), especially CO_2_ [118]. Effects of such impacts have lead to decline of biodiversity in reefs of Brazil and of the world, through increase of sea temperature and ocean acidification [11]. Local impacts boost these effects through higher sedimentation, multiple biological invasions, bleaching, coral diseases and, consequently, loose of diversity on reef environments [11, 141, 142, 143]. Such impacts are frequently related to disorganized urban growth, pollution, messy tourism practices and overfishing [144, 145, 146]. In the literature *M*. *harttii* used to be described as forming extensive bands on coastal reefs, showing colonies usually up to 1 m in diameter [120]. Currently, this is a rare scenario for most of these reefs, which often have a low coral coverage, not corresponding the descriptions of the 1970’s.

Environmental changes have triggered reorganizations in reef ecological relationships, zonation and dominance, in processes also called *phase-shifts* [147]. In most reefs, for example, scleractinian dominance have been replaced mainly by macroalgae [119], octocorals [148], sponges [149] and/or zoanthids [150, 151, 152], the latter is the case of the Brazilian reefs [124]. In these reefs, *M*. *harttii* is also threatened by the dominance of invasive species, such as *Tubastraea* spp. [153], which further compromises its resilience of shallow reefs.

The accelerated loss of biodiversity and habitats is one of the worst crisis of the present time, as evidenced by the ever increasing species red lists. All current and future scenarios showed herein alert for the relevance of the endemism and the role of *M*. *harttii* as a reef builder in Brazilian reefs. Currently, the species is classified as “in risk of extinction” [19], and the perspective of reduction of suitable shallow areas highlight the urgency of priority conservation measures. Future environmental politics, therefore, must focus not only in the recovery of coastal populations, but also on the conservation of mesophotic coral ecosystems (MCE’s). Despite being less affected by climate changes, MCE’s are impacted by human activities, such as fisheries, mining and drilling [136, 154] and measures to protect deeper ecosystems should be prioritized in environmental policies for marine conservation, especially in Brazil.

### Conclusions

This research showed the efficiency of SDMs to predict areas with potential habitat to *M*. *harttii* in the present scenario and in two future scenarios for the end of the 21st century. The results showed a significant reduction of the area with potential habitat for the species. The largest area of the FPH for *M*. *harttii* is concentrated in deeper waters, especially within the Abrolhos bank, one of the most important areas for biodiversity conservation in the South Atlantic [141]. Despite having several protected areas, Abrolhos still suffers anthropic impacts, which tend to increase in the next decades, by activities such as port expansion, overfishing, mining and oil/gas extraction. Therefore, it is essential to ensure viable refuges not only for the endangered *M*. *harttii*, but for all Brazilian coral species, given the expected climate change scenarios. Thus, an expansion of protective measures focused on mesophotic reefs, especially in the Abrolhos Bank, is essential and urgent.

## Supporting information

S1 Appendix Occurrence records used to generate maps of Current Potential Habitat and Future Potential Habitat (modeling records) and records to evaluation the models (evaluation records). Georeference (latitude and longitude), source and author of occurrence records used to generate the CPH and FPH models and for evaluation the models.
(XLSX)

S2 Appendix Principal Component Analysis and Pearson correlation. Result of the analyzes used for the selection of environmental variables.
(XLSX)

S3 Appendix Maxent output of Current Potential Habitat and Future Potential Habitat. Maxent output with values of threshold, AUC, percentage of the predicted area and number of occurrences used to generate the Current Potential Habitat and Future Potential Habitat model.
(XLSX)

S4 Appendix Results of Ecological Niche Models evaluation (ENMeval). Output from the ENMeval package of the model used for CPH (best model) and general values of 14 for each set of variables.
(XLSX)

S5 Appendix Output of the ntbox used to evaluate the AUCratio of CPH and FPH. Values of AUCratio for a AUCrandon (at level of 0.5) and the AUCatual (calibrating 5% of omission and 1000 bootstrap interactions).
(XLSX)

S6 Appendix Unpublished work. Cordeiro, RTS; Amaral, FMD. Ocorrência de cnidários construtores de recifes em ambientes de profundidade no Nordeste do Brasil. In: Abstracts of XIV Congreso Latinoamericano de Ciencias del Mar, 2011, Balneário Camboriú - SC, Brazil.
(PDF)
