# Supplementary material for: Modeling impacts of climate change on the potential habitat of an endangered Brazilian endemic coral: Discussion about deep sea refugia
Source: PLoS One. 2019 May 21;14(5):e0211171. doi: 10.1371/journal.pone.0211171 (PMC6529159; doi:10.1371/journal.pone.0211171)
Supplement: S6 Appendix — Cordeiro, RTS; Amaral, FMD. Ocorrência de cnidários construtores de recifes em ambientes de profundidade no Nordeste do Brasil. In: Abstracts of XIV Congreso Latinoamericano de Ciencias del Mar, 2011, Balneário Camboriú - SC, Brazil. (PDF) [file pone.0211171.s006.pdf]

ResearchGate

See discussions, stats, and author profiles for this publication at: https://www.researchgate.net/publication/267717033

OCORRÊNCIA DE CNIDÁRIOS CONSTRUTORES DE RECIFES EM AMBIENTES DE PROFUNDIDADE NO NORDESTE DO BRASIL

Article

CITATION
1

READS
251

2 authors:

Ralf Cordeiro
Universidade Federal Rural de Pernambuco
20 PUBLICATIONS 50 CITATIONS

SEE PROFILE

Fernanda M D Amaral
Universidade Federal Rural de Pernambuco
55 PUBLICATIONS 383 CITATIONS

SEE PROFILE

Some of the authors of this publication are also working on these related projects:

Project Diversity of octocorals from West Atlantic Ocean View project

Project Diversity of black corals from South Western Atlantic Ocean View project

All content following this page was uploaded by Ralf Cordeiro on 19 January 2015.

The user has requested enhancement of the downloaded file.

OCORRÊNCIA DE CNIDÁRIOS CONSTRUTORES DE RECIFES EM AMBIENTES DE PROFUNDIDADE NO NORDESTE DO BRASIL

Ralf Cordeiro e Fernanda Amaral[2]

Introdução

O Filo Cnidaria tem cerca de 20 representantes no Brasil que podem contribuir ativamente para a construção de recifes de coral, desses, a maior parte é de corais escleractínios e o restante de hidróides calcários [1]. Todos estes, no entanto, dependem de uma relação de simbiose com dinoflagelados fotossintetizantes para o desempenho perfeito do papel de construtores. Estes simbiontes pertencem ao gênero *Symbiodinium* e são mais conhecidos como zooxantelas [2]. A partir desse contexto, temos duas divisões genéricas: cnidários zooxantelados e azooxantelados [3,4]. Essa denominação implica numa série de fatores ecológicos, bióticos e abióticos, inerentes à manutenção da relação coral x zooxantela, tais como temperatura e intensidade luminosa, que são influenciados diretamente pela profundidade.

Uma vez que zooxantelas são fotossintetizantes, é de se inferir que os organismos aos quais elas se associam se distribuam em locais rasos, de pouca sedimentação e de luminosidade e temperatura elevadas, ou seja, ambientes recifais. Por conseguinte, todos os cnidários construtores de recifes de águas rasas são zooxantelados [1]. E, em se tratando desses ecossistemas, é essencial que esses organismos sejam bastante tolerantes a grandes variações ambientais. No entanto, nos últimos anos, a frágil e relativa estabilidade mantida nos ambientes recifais vêm sendo ameaçada por alterações ambientais resultantes da ação antrópica, como o aquecimento global, em grande escala, e a eutrofização de ambientes costeiros, como degradações mais pontuais [5].

Mais recentemente, pesquisas com finalidades comerciais e científicas têm sido realizadas, visando uma maior e melhor exploração de recursos em águas profundas ao redor do mundo, uma vez que seus habitantes são menos afetados por mudanças climáticas (mesmo sendo vítimas de degradação via atividades pouco regulamentadas) [3,6]. No Brasil, apesar de os esforços nesse sentido ainda serem poucos [4,7], algumas expedições oceanográficas, resultantes de parcerias do Governo Federal, Marinha do Brasil e instituições de pesquisa têm contribuído para uma melhor compreensão da biodiversidade desses ambientes.

Tendo em mente a estabilidade desses ambientes profundos, assim como a necessidade de preservação de corais zooxantelados para a futura construção e manutenção do arcabouço de ecossistemas recifais em águas rasas, o objetivo do presente trabalho é, portanto, reportar a ocorrência de corais e hidróides calcários zooxantelados em ambientes de profundidade no nordeste do Brasil.

Material e métodos

Os exemplares estudados foram coletados por ocasião da expedição REVIZEE (NEIII), realizada de maio a julho de 1998, e depositados na coleção de invertebrados bentônicos do Departamento de Oceanografia da Universidade Federal de Pernambuco.

A identificação, assim como algumas análises morfológicas e contagem de caracteres, foi realizada de acordo com literatura pertinente [8,9].

Resultados e Discussão

A seguir, são descritas seis espécies de corais zooxantelados, juntamente com duas espécies de hidróides calcários também zooxanteladas. A temperatura nas estações de coleta teve uma média de 27,87ºC ± 1,48 e a salinidade 36,33 ± 0,31.

Foram coletados cerca de 20 ramos do hidróide *Millepora alcicornis* Linnaeus, 1758 (Fig. 1C) não havendo registro de incrustações de eixo de gorgônias mortas, diferindo do que é citado por Amaral et al. [10], as colônias, quando não arborescentes, acompanhavam a morfologia do substrato. *Millepora braziliensis* Verrill, 1868, espécie endêmica do litoral e ilhas oceânicas brasileiras, semelhante a *M. alcicornis* apresentou colônias incrustantes, no entanto, as colônias analisadas foram maciças, com até 10 cm de altura. Apesar de essas espécies terem ocorrência citada em profundidades de até 40 metros [10], a maioria dos exemplares estudados foram coletados em profundidades entre 50 e 60 m (Tab. 1).

O coral *Agaricia agaricites* (Linnaeus, 1758) (Fig. 1D) foi coletado a 55 m de profundidade. Uma das colônias coletadas não apresentou estrutura de fixação ao substrato, o que é atípico nesta espécie, por ser frequentemente incrustante [11].

Hetzel & Castro [11] citam que *Mussismilia harttii* (Verrill, 1868) (Fig. 1B) pode ocorrer em profundidades

1. Graduando do curso Bacharelado em Ciências Biológicas, Universidade Federal do Rural de Pernambuco. Rua Dom Manuel de Medeiros, Dois irmão, s/n, Recife, PE, CEP 52171-900. E-mail: ralfts@hotmail.com
2. Professora Associada do Departamento de Biologia, Área de Zoologia, Universidade Federal do Rural de Pernambuco. Rua Dom Manuel de Medeiros, Dois irmão, s/n, Recife, PE, CEP 52171-900.

que variam de 2 a 30 m, sendo eventualmente encontrada a 80 m. Semelhante a esta afirmação, os exemplares de *M. harttii* foram coletados em duas estações a 70 m (Tab. 1), o que já é um ambiente pouco propício ao desenvolvimento de corais zooxantelados. Os quase 30 espécimes e/ou fragmentos analisados tinham morfologia da variedade laxa, segundo definida por Laborel [12].

As colônias e/ou fragmentos de *Siderastrea stellata* Verrill, 1868 não ultrapassaram 10 cm de diâmetro. Esta espécie é endêmica do Brasil [10] e os exemplares estudados foram sempre incrustantes, mas raramente de grande espessura.

Os corais *Porites astreoides* Lamarck, 1816 e *P. branneri* Rathbun, 1888, coletados a aproximadamente 55 e 57 m, respectivamente, apresentaram-se danificados. Não se pode afirmar, no entanto, que *P. astreoides* colonize substratos nessa profundidade, uma vez que só foi obtido um único fragmento desta espécie, podendo tratar-se de um exemplar rolado de regiões mais rasas.

Colônias esféricas, maciças e plocóides são características do coral *Montastraea cavernosa* (Linnaeus, 1767) (Fig. 1A), que foi coletado em profundidades de até 55 m. Este coral, juntamente com *M. harttii* e *M. alcicornis*, é um dos registros mais importantes do presente estudo, uma vez que estas espécies estão entre os principais construtores de recifes brasileiros. Sua ocorrência em ambientes de profundidade já foi reportada em profundidades semelhantes [6], no entanto, ainda necessita de maiores esclarecimentos.

Uma vez tomada a consciência do estado de pouco conhecimento sobre os recursos desses ambientes, assim como de sua biodiversidade, faz-se necessária a ampliação e intensificação de linhas de pesquisa nessa área.

Agradecimentos

Ao Museu de Invertebrados Bentônicos da UFPE, pelo apoio.

Referências

[1] LEÃO, Z. M. A. N.; KIKUCHI, R. K. P. & TESTA, V., 2003. Corals and coral reefs of Brazil. In: CORTEZ, J. (ed.). *Latin American Coral Reefs*, p.9- 53.

[2] DAVY, S.K. & TURNER, J.R. 2003. Early Development and Acquisition of Zooxanthellae in the Temperate Symbiotic Sea Anemone *Anthopleura ballii* (Cocks). *Biol. Bull.* 205: 66–72.

[3] KITAHARA, M. V. 2007. Species Richness and Distribution of Azooxanthellate Scleractinia in Brazil. Bulletin of Marine Science, 81(3): 497–518.

[4] CAIRNS, S. D. 2007. Deep-water corals: an overview with special reference to diversity and distribution of deep-water scleractinian corals. *Bulletin of Marine Science*, 81(3): 311–322.

[5] LEÃO, Z. M. A. N. & KIKUCHI, R. K. P. 2005. A relic coral fauna threatened by global changes and human activities, Eastern Brazil. *Marine Pollution Bulletin*, 51: 599–611.

[6] VENN, A. A.; WEBER, F. K.; LORAM, J. E. & JONES, R. J. 2009. Deep zooxanthellate corals at the high latitude Bermuda Seamount. *Coral Reefs*, 28:135.

[7] KITAHARA, M. V. 2006. Novas ocorrências de corais azooxantelados (Anthozoa, Scleractinia) na plataforma e talude continental do sul do Brasil (25-34º S). *Revista Biotemas,* 19 (3): 55-63.

[8] VAUGHAN, T. W. & WELLS, J. W. 1943. Revision of the suborders, families and genera of the Scleractinia. *Special Papers of Geological Society of America*, 44: 1-363.

[9] AMARAL, F. M. D.; STEINER, A. Q.; BROADHURST, M. K. & CAIRNS, S. D. 2008. An overview of the shallow-water calcified hydroids from Brazil (Hydrozoa: Cnidaria), including the description of a new species. *Zootaxa*, 1930: 56–68.

[10] AMARAL, F. M. D.; HUDSON, M.M.; STEINER, A. Q. & RAMOS, C.A.C. 2007. Corals and calcifed hydroids of the Manuel Luiz Marine State Park (State of Maranhão, Northeast Brazil). *Biota Neotropica*, 7(3): 1-9.

[11] HETZEL, B. & CASTRO, C. B. 1994. *Corais do sul da Bahia*. Rio de Janeiro: Nova Fronteira, 189 pp.

[12] LABOREL, J. L. 1969. Les peuplements de madreporaires des côtes tropicales du Brésil. *Ann. Univ. d'Abidjan*, II(3): 39-47.

Tabela 1. Listagem de espécies de corais e hidróides calcários estudados, assim como respectivas coordenadas e profundidades de coleta.

Espécies	Coordenadas	Profundidade (m)
Millepora alcicornis Linnaeus, 1758	1º54'06" S 37º49'44" W	55,05
Millepora braziliensis Verrill, 1868	1º29'13" S 38º41'22" W	54,00
Agaricia agaricites (Linnaeus, 1758)	1º54'06" S 37º49'44" W	55,05
Mussismilia harttii (Verrill, 1868)	3º48'27" S 33º40'59" W	70,00
Siderastrea stellata Verrill, 1868	4º15'34" S 33º14'34" W	49,20
Porites astreoides Lamarck, 1816	1º29'13" S 38º41'22" W	54,00
Porites branneri Rathbun, 1888	1º37'42" S 38º09'56" W	56,70
Montastraea cavernosa (Linnaeus, 1767)	3º49'53" S 34º43'46" W	54,60

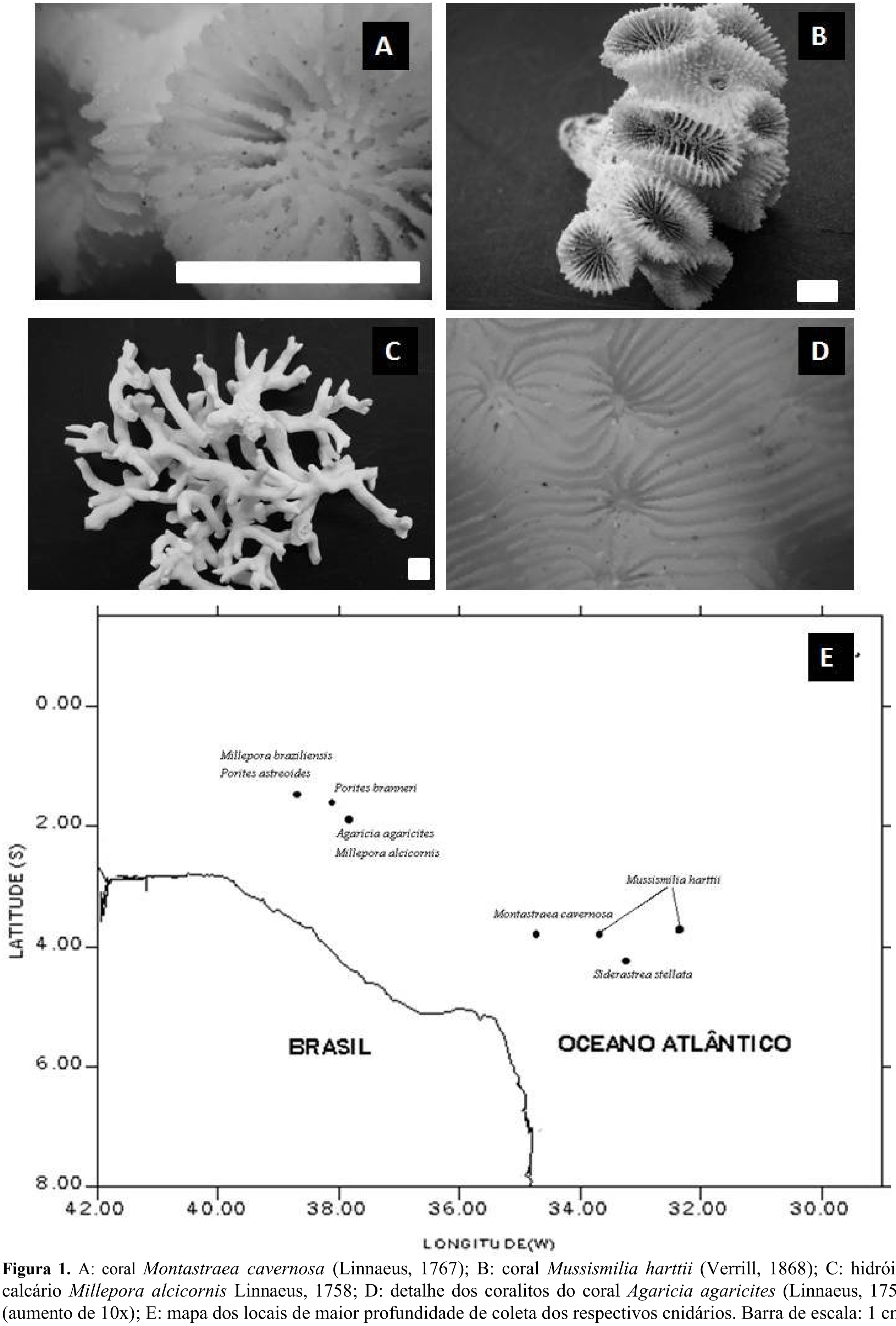

Figura 1. A: coral *Montastraea cavernosa* (Linnaeus, 1767); B: coral *Mussismilia harttii* (Verrill, 1868); C: hidróide calcário *Millepora alcicornis* Linnaeus, 1758; D: detalhe dos coralitos do coral *Agaricia agaricites* (Linnaeus, 1758) (aumento de 10x); E: mapa dos locais de maior profundidade de coleta dos respectivos cnidários. Barra de escala: 1 cm.

View publication stats
